# Breast Reconstruction using the Anterior Approach Scarless Latissimus Dorsi Muscular flap: A Single Center Retrospective Study

**DOI:** 10.1016/j.jpra.2024.03.004

**Published:** 2024-03-21

**Authors:** Andrea Loreti, Edoardo Bruno, Ornella Abate, Floriana Arelli, Diana Spallone, Massimo La Pinta, Tiziana Matropietro, Elisabetta Ponti, Laura Broglia, Leopoldo Costarelli, Paola Scavina, Diego Ribuffo, Lucio Fortunato

**Affiliations:** a Plastic and Reconstructive Surgery Division, Azienda Ospedaliera San Giovanni-Addolorata, Via Dell'Amba Aradam 8, Rome, Italy; b Department of Surgery “P.Valdoni,” Unit of Plastic and Reconstructive Surgery, Policlinico Umberto I, Sapienza University of Rome, via dei Latini 33, 00185 Rome, Italy; c Breast Unit, Azienda Ospedaliera San Giovanni-Addolorata, Via Dell'Amba Aradam 8, Rome, Italy; d Radiation Oncology Division, Azienda Ospedaliera San Giovanni-Addolorata, Via Dell'Amba Aradam 8, Rome, Italy; e Breast Radiology Division, Azienda Ospedaliera San Giovanni-Addolorata, Via Dell'Amba Aradam 8, Rome, Italy; f Pathology Division, Azienda Ospedaliera San Giovanni-Addolorata, Via Dell'Amba Aradam 8, Rome, Italy; g Oncology Division, Azienda Ospedaliera San Giovanni-Addolorata, Via Dell'Amba Aradam 8, Rome, Italy

**Keywords:** Anterior approach scarless latissimus dorsi flap, Autologous breast reconstruction, Latissimus dorsi muscle

## Abstract

**Introduction:**

Scarless latissimus dorsi (LD) flap is a breast reconstruction technique, which allow to cover the lower pole of implant with a large portion of the LD muscle without skin paddle; it represents a surgical solution that transpose vascularized tissue avoiding the failure of breast reconstruction, following necrosis of mastectomy skin flaps.

**Material and Method:**

A retrospective review of patients undergoing immediate or delayed breast reconstruction using scarless LD flap reconstructions was performed. Clinical data obtained from follow-up visits were recorded. To evaluate breast shape contentment and patient satisfaction, the patients were requested to answer the Breast-Q, version 2.0 reduction module postoperative scales questionnaire at the 12-month follow-up.

**Results:**

We performed 19 scarless LD flap reconstructions between September 2019 and June 2022. The surgical time in average (considering minutes ± SD) was 130 (±15) minutes. The aesthetic assessment was good/excellent in 83% of patients. This was statistically significant (P=0.0).

**Conclusions:**

The scarless LD flap reconstruction is a valid and reliable solution, which has the advantage to reduce the risk of exposed prosthesis if native skin necrosis occurs.

## Introduction

The evolution of breast reconstruction over the last few years followed the trend of preserving the native skin of the breast to the maximum extent possible.^1^^,^^2^^,^^3^ There has been an increase in the use of fat grafting in revision surgery,^4^ and since 2006, the use of acellular dermal matrices (ADMs) and synthetic meshes has been introduced to cover the lower pole of the implant.^5^, ^6^, ^7^, ^8^, ^9^, ^10^, ^11^, ^12^, ^13^

Among the main problems associated with the use of ADMs, there are the high costs and the high rate of complications, such as seromas, infections, and necrosis of mastectomy flaps with failure of breast reconstruction.^14^^,^^5^

The use of the scarless latissimus dorsi (LD) muscle flap to cover the lower pole of the breast prosthesis represents a surgical alternative to ADM or synthetic mesh in presence of poor soft tissue coverage after primary breast reconstruction failure, especially in irradiated patients; the scarless LD flap with anterior approach using the mastectomy scar allows to harvest a large portion of the LD muscle without adding further scars or using endoscopic technique.^15^^,^^16^

## Materials and methods

### Objectives

The primary endpoint was to assess the safety and aesthetic outcomes of patients treated with anterior approach scarless muscular LD flap for breast reconstruction after mastectomy.

### Patients

For this retrospective analysis, patients who underwent unilateral nipple-sparing and skin-sparing mastectomies with anterior approach scarless LD flap reconstruction, treated between September 2019 and June 2022, were retrospectively studied. The patients have undergone surgery at the Department of Plastic Surgery of San Giovanni-Addolorata Hospital, Rome, Italy. All the surgical procedures were performed by the author of this article (Loreti A. MD). Exclusion criteria were pediatric patients (0-18 yr) and the absence of follow-up.

### Surgical Technique

Preoperatively, with the arm raised, the anterior border of the LD muscle is drawn, considering that it extends from the posterior edge of the axilla down to the iliac crest.

**Figure 1a-c:**
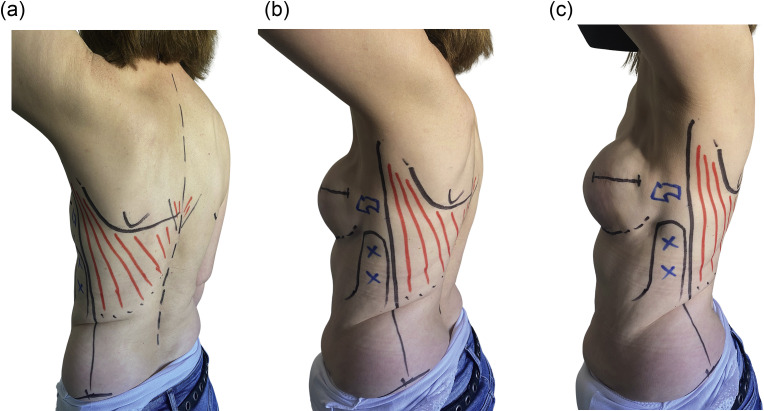
Preoperative drawing performed with the patient's arm elevated. The anterior border of the muscle is marked by drawing a line from the posterior border of the axilla toward the iliac crest. The inferior segment of the trapezius, as it overlaps the upper medial border of the latissimus dorsi muscle and inframammary fold are marked. The skin portion that must not be detached from the underlying tissues laterally to the breast is highlighted.

The inferior segment of the trapezius is drawn as it overlaps the upper medial border of the LD as a reminder of this anatomic relationship. The inframammary fold and the area at the lateral margin of the breast in which dissection must be avoided are also marked (Fig. 1a-c).

**Figure 2:**
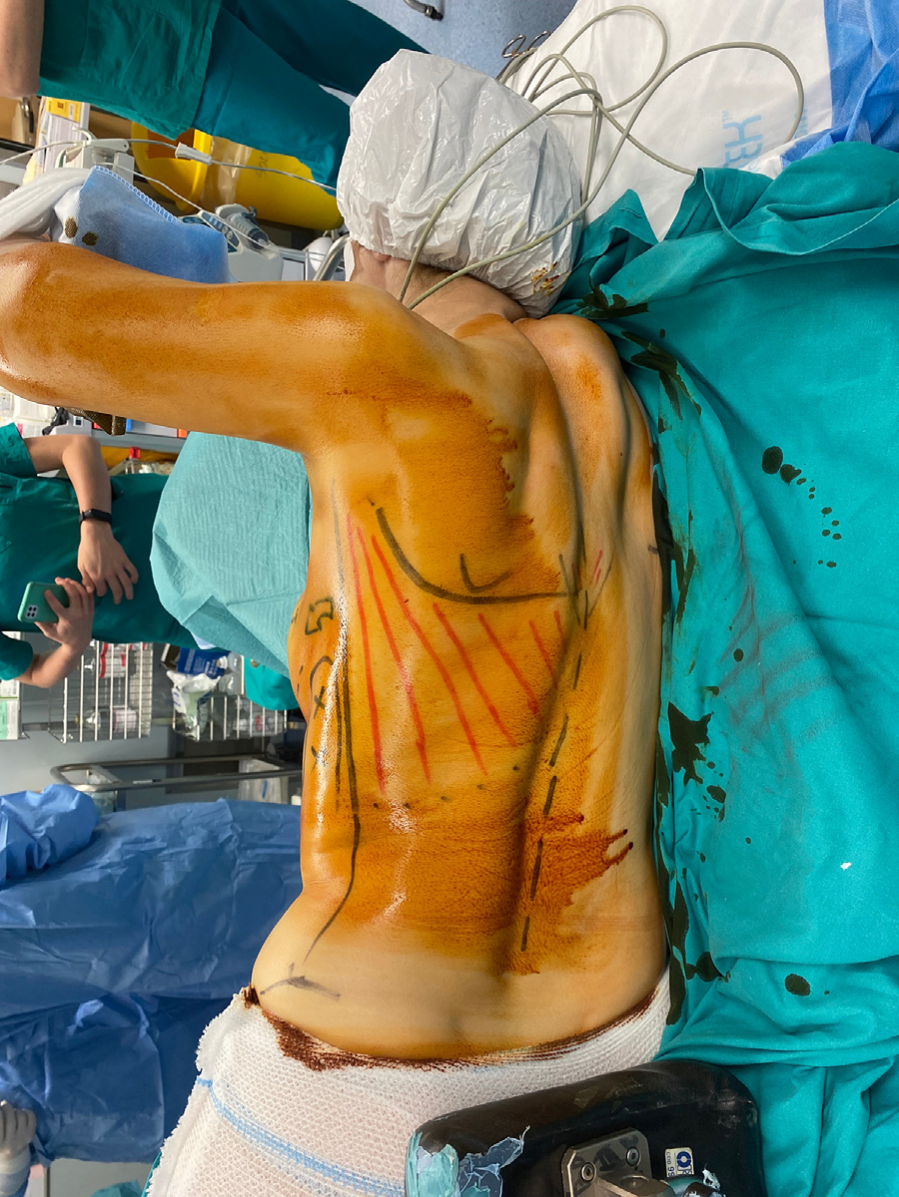
Patient positioning.

**Figure 3:**
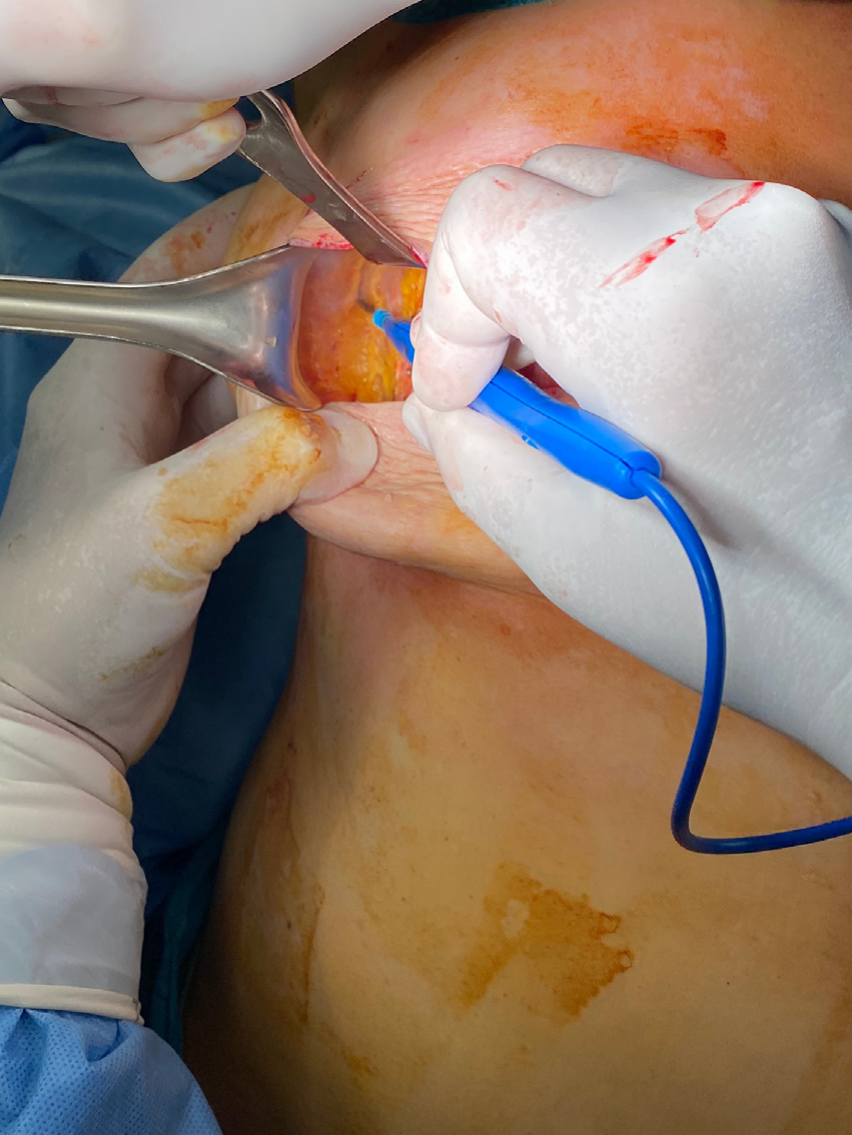
Skin dissection cranial and lateral to the breast from the mastectomy incision.

**Figure 4:**
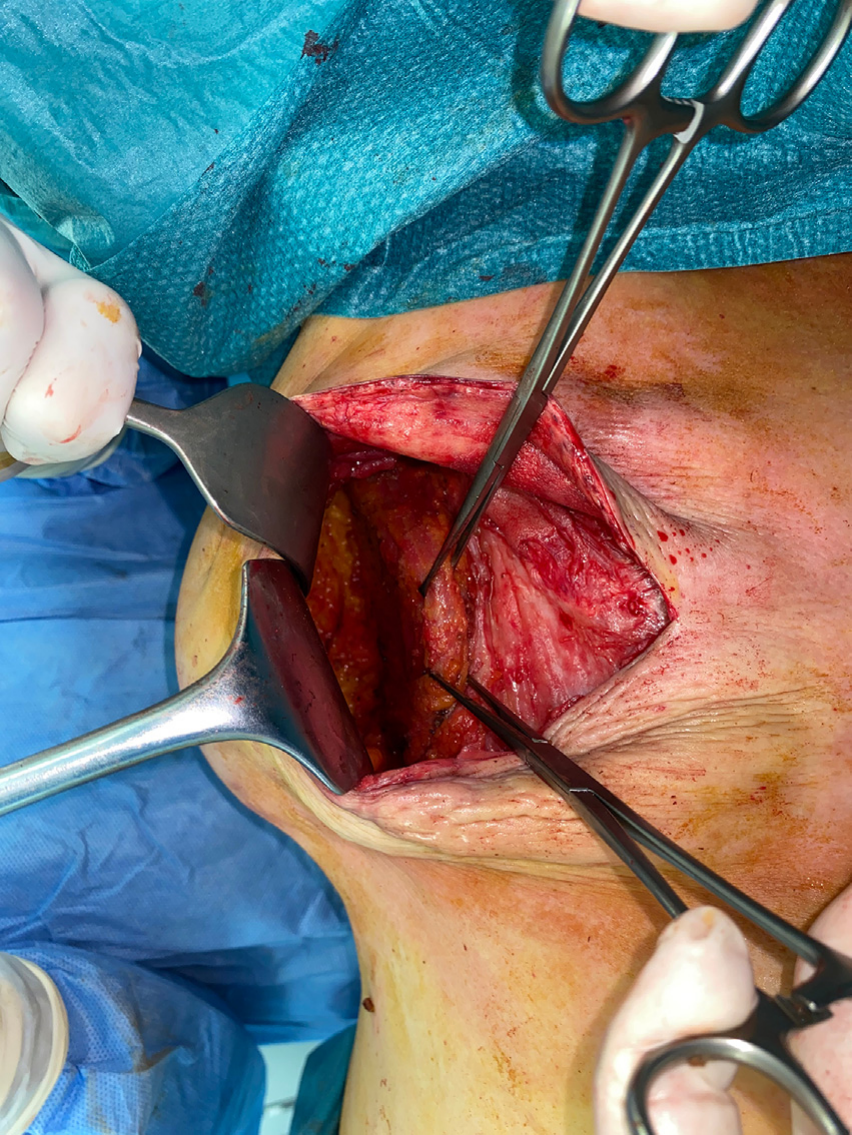
Anterior border of the latissimus dorsi muscle

**Figure 5:**
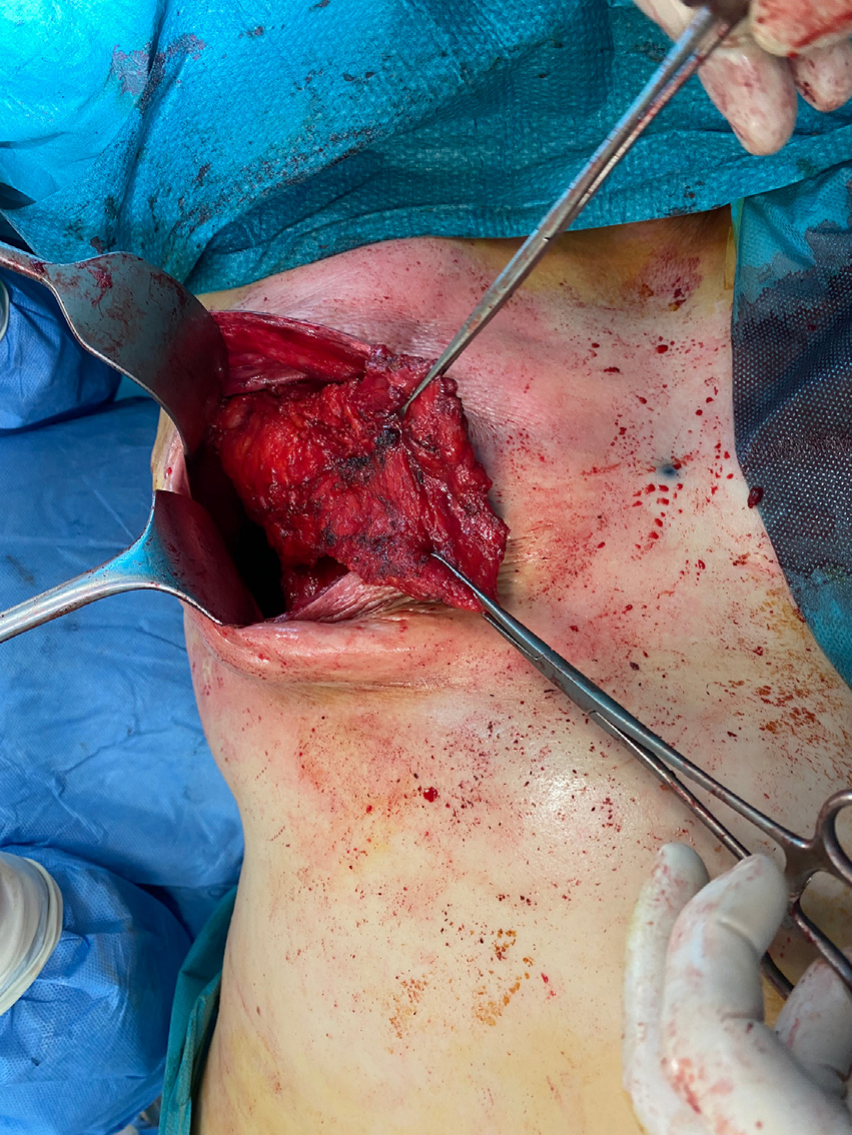
Muscle rotated and stretched across the midline toward the breast after its tendon dissection.

The patient is placed in a lateral decubitus position (Fig. 2).The scarless LD flap is harvested from an anterior approach through the mastectomy incision (Fig. 3); skin flaps are retracted laterally to allow the identification of the lateral edge of the LD muscle (Fig. 4). At this point, using a fiber optic retractor, the dissection proceeds superiorly over the muscle fascia through the medial edge of the LD muscle; subsequently, the deep surface of muscle is detached from the thoracic wall. Dissection procedure should proceed as dorsally as possible. The pedicle needs to be identified, and a careful blunt dissection must be carried out around the tendon. The inferior scapula fat pad is the landmark for division of the muscle. Once the muscle is released inferiorly, it is used for counter traction while dissecting cranially. The tendon can be dissected to its point of origin, allowing for improved rotation and a gradual stretch to reach the sternal line (Fig.5, Video 1). If needed, the tendon can be divided, preserving the vascular pedicle of the flap to increase the arc of rotation.

**Figure 6:**
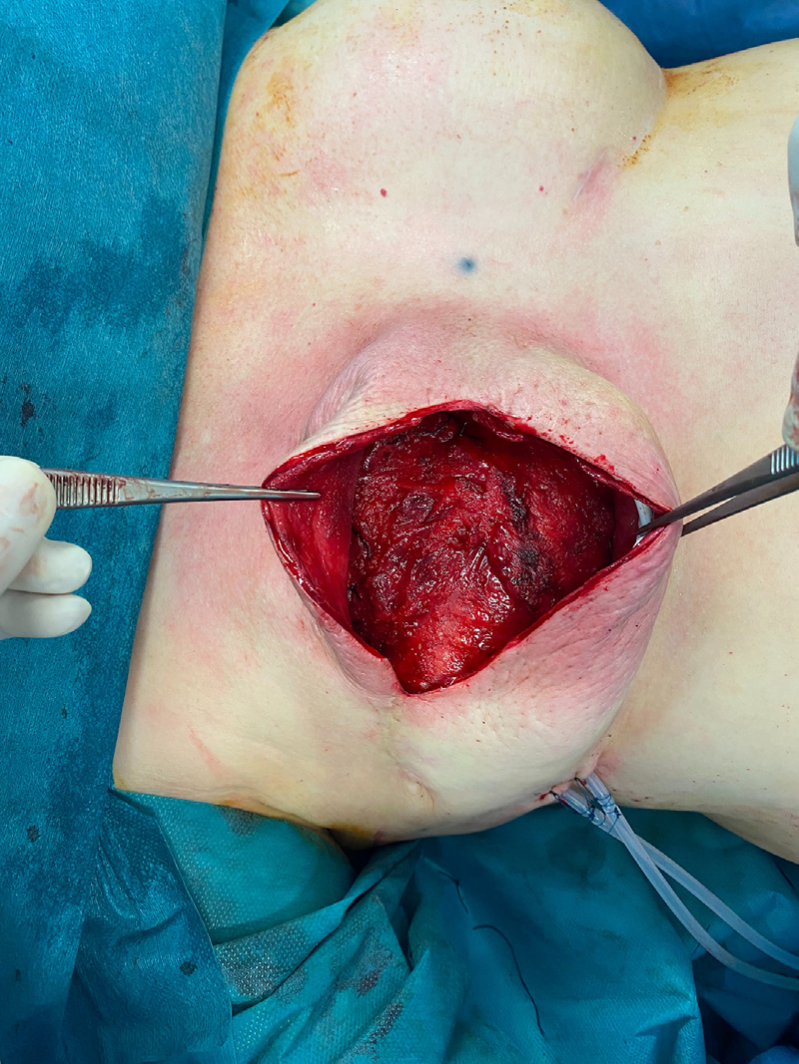
Prosthetic implant covered superiorly by the pectoralis major and latissimus dorsi muscles on the lower pole.

An expansion prosthesis or a definitive implant are inserted covered in its lower pole by LD muscle flap and in its upper pole by pectoral major muscle (Fig. 6). The LD is secured to the lower edge of pectoralis muscle and sutured to the periosteal rib at the site of the future inframammary fold with 2.0 Vicryl sutures. One suction drain is placed in the donor side and one in the pocket.

### Demographics, Clinical, Surgical, and Pathological Data

Information regarding age and body mass index at the time of the operation, the presence of risk factors such as diabetes or smoking, preoperative assessment of patients’ breast volume using the BREAST-V, timing of breast reconstruction, type of breast cancer, type of surgery (including potential axillary dissection during the initial surgery), adjuvant radiochemotherapy, and follow-up status (including follow-up duration) was systematically gathered for all participants in the study. Additionally, data were compiled on hospitalization duration, surgical duration, volume of the inserted expander, and permanent implant for any complications. The patients were followed up at the outpatient clinic at 2- and 4-weeks, and 3-, 6-, and 12-months postoperatively. Data concerning outcomes were retrieved from mortality registries, outpatient visits, and radiological follow-up.

### Ethical considerations

The study protocol underwent review and was approved by institutional ethics committees before commencement. The study adhered to the principles of the Helsinki Declaration. All requisite clinical data were documented in a computerized database. Patients who were still alive at the time of the study were informed about it and none objected to their inclusion.

### Statistical analysis

Quantitative variables were described with median and range [min-max], while qualitative variables were described with numbers and percentages. Chi square or Fisher exact tests were used to compare categorical variables. Progression free survival and overall survival were assessed using the Kaplan–Meier estimator and compared using a log-rank test. For all tests, a two-tailed P value less than or equal to 0.05 was considered statistically significant.

## Results

Nineteen unilateral breast reconstruction procedures with scarless LD flap were performed between September 2019 and June 2022; 4 procedures were carried out concurrently with the mastectomy, while 15 were performed in a delayed surgical time. Thirteen reconstructions were performed in patients who underwent nipple-sparing mastectomy, while six in patients who underwent skin-sparing mastectomy. Seven cases of scarless LD reconstruction were performed in patients who previously underwent mastectomy and prosthesis reconstruction and developed severe capsular contracture.

The average age of the 19 patients was 45 years, with a range of 36 to 59 years. In 5 cases of reconstruction with scarless LD flap, a defined prosthesis was placed, and in the remaining 14 cases, an expander was placed and subsequently replaced with a permanent implant.

The mean hospitalization time (days ± SD) was 3.7 (±1.0) days with a range of 3-6 days, while the postoperative mean stay (days ± SD) was 2.7 (±1.0) days. The mean follow-up (months ± SD) was 19 ± 7.8 months with a range of 6-38 months.

The mean surgical time (minutes ± SD) was 130 (±15) minutes and the average time between the mastectomy and flap (days ± SD) was 955 ± 114 days.

The mean volume of inserted expander was 384 ± 145 (150–750) mL and the average permanent implant size (mean ± SD) was 317 cm^2^.

Overall, 84,21% of the patients before reconstruction surgery with the scarless LD flap had radiotherapy, while 10.52% of patients had radiotherapy after scarless LD flap procedure. There were no major complications during or after surgeries and three patients developed at least one complication (Tab 2).The overall complication rate was 15.7%: 2 seromas, which were treated with needle aspiration and compressive dressing, and 1 case of native breast skin necrosis that was effectively managed conservatively, yielding excellent results. The overall rate of reconstruction failure with the scarless LD flap with prosthesis was 0/19.

Among the 19 procedures, CC Baker grade III and IV was recorded in only 2 cases (10.52%) (Table 1). All the patients with a diagnosis of severe CC underwent revisional surgery. The relation between CC and patient and tumor characteristics is described in Table 1.

**Table 1:** Demographics of patients who underwent breast surgery with anterior approach scarless latissimus dorsi flap reconstruction DCIS, ductal carcinoma in situ; LCIS, lobular carcinoma in situ.

CHARACTERISTICS		Overall	CC Baker grade I-II	CC Baker grade III-IV
			17 (89.47%)	2 (10.52%)
Age (years, mean ± SD)		45.73 ± 6.18	46.47 ± 6.12	39.50 ± 0.70
BMI (kg/m^2^, mean ± SD)		23.73 ± 2.42	23.88 ± 2.47	22.50 ± 2.12
Breast V (mean ± SD)		402.72 ± 32.55	400.17 ± 63.45	424.36 ± 63.45
Smoking/Diabetes	No	16 (84.21%)	15 (78.94%)	1 (5.26%)
	Yes	3 (15.78%)	2 (10.52%)	1 (5.26%)
Timing of breast reconstruction	Immediate	4 (21.05%)	3 (15.78%)	1 (5.26%)
	Delayed	15 (78.94%)	14 (73.68%)	1 (5.26%)
Type of cancer	DCIS	10 (52.63%)	10 (52.63%)	0
	LCIS	3 (15.78%)	2(10.52%)	1 (5.26%)
	Multifocal	6 (31.57%)	5 (26.31%)	0
Axillary lymph node dissection	No	11 (57.89%)	11 (57.89%)	0
	Yes	8 (42.10%)	6 (31.57%)	2 (10.52%)
Radiotherapy	No	2 (10.52%)	2 (10.52%)	0
	Preoperative	16 (84.21%)	14 (73.68%)	2 (10.52%)
	Postoperative	2 (10.52%)	2 (10.52%)	0
Systemic treatment	No	7 (36.84%)	6 (31.57%)	1 (5.26%)
	Preoperative	3 (15.78%)	2 (10.52%)	1 (5.26%)
	Postoperative	9 (47.36%)	9 (47.36%)	0

**Table 2:** Overall patient complications.

COMPLICATIONS	
Failure of reconstruction	0 (0%)
Capsular contracture	0 (0%)
Hematoma	0 (0%)
Implant exposure	0 (0%)
Skin necrosis	1 (5.2%)
Seroma	2 (10.5%)
Infection	0 (0%)

Patient satisfaction was assessed using the standardized Breast-Q questionnaire (Table 3) and aesthetic outcomes were assessed, reporting 83% good to excellent results.

**Table 3:** Results of the BREAST-Q questionnaire.

BREAST-Q Scales	Minimum	Maximum	Mean	Std. Deviation
Psychological well-being	36	100	72.56	21.83
Sexual well-being	36	100	68.84	25.11
Physical well-being	29	100	79.21	22.13
Satisfaction with surgical results	20	100	78.13	19.45
Satisfaction with information	28	100	81.78	19.66
Satisfaction with the surgeon	24	100	79.64	18.94
Satisfaction with the surgical team	0	100	87.56	19.21
Satisfaction with the hospital	0	100	69.78	19.83

## Discussion

Breast reconstruction in presence of primary surgery complications or failure, poor soft tissue coverage, and irradiated tissue is the main challenge. In these cases, the goal of the breast reconstruction is reaching good results with less morbidity and minimizing scars, surgical, and hospitalization time. Breast reconstruction with anterior approach scarless LD muscle flap can achieve these goals in selected cases.^17^, ^18^, ^19^

The traditional myocutaneous LD flap breast reconstruction imports a large skin paddle from the back causing an evident scar and often resulting in a breast skin color mismatch, reducing aesthetic outcome.

Instead using the anterior approach scarless LD muscle flap allows to obtain good pliability and laxity of the reconstructed lower breast pole.

One limitation of the “scarless” technique is the inability to monitor the flap in the traditional manner. However, given the extremely low risk of flap compromise, the advantages, such as the absence of scarring and shorter operative time, outweigh the described disadvantage. The flaps were clinically monitored through evaluation of swelling and tissue turgor. In case of suspected vascular compromise or to exclude the presence of hematomas or seromas, we performed duplex Doppler ultrasound evaluation. In all cases of two-stage breast reconstruction, the viability of the LD muscle flap was confirmed during the second stage. Additionally, none of the single-stage reconstruction cases showed any clinical signs indicating vascular compromise of the flap.

Moreover, the tissue fibrosis, atrophy, and inhibition of healing mechanisms^20^ makes breast reconstruction after postmastectomy radiotherapy difficult and unsafe with a high rate of capsular contracture.

Heterologous breast reconstructions performed after radiotherapy have a relatively high failure rate. Cordeiro et al.^21^ presented a large series of two-stage implant-based breast reconstructions in which the reconstruction failure rate in irradiated patients was more than 10-fold greater than that in non-irradiated patients. A recent literature review^22^ reported that adjuvant radiotherapy resulted in an higher reconstruction failure rate in prosthetic breast reconstruction. Lastly, a relevant meta-analysis by Momoh et al.^23^ reported that reconstruction failure rates were extremely similar, at 19% and 20% for patients with previous histories of radiotherapy and postoperative irradiation, respectively. Among the issues related to the alternative solution of total submuscular tissue expansion is that dissection to serratus anterior muscle off the ribs inferolaterally, for the coverage of the implant, produces considerable postoperative pain; that together a restriction of the lower pole with a tendency for the expander to migrate high and even rotate, which makes the result of tissue expansion unpredictable. The anterior approach scarless LD muscle flap avoids these problem, because it does not require the painful periosteal stripping associated with serratus anterior harvest and produces less restriction of the lower pole.^12^^,^^24^ Furthermore, the muscular transposition allows vascularization of the irradiated skin; this helps skin regeneration, improving the quality and trophism of the skin.^25^ The cosmetic outcome is comparable with those of the other methods of contouring the lower pole, in absence of a patch effect, and eventually the risk of reconstructive failure is dramatically decreased.^26^

Other procedures have been described to ensure implant coverage, including lipofilling and the use of ADM. Ribuffo et al. proposed protective lipofilling in cases of irradiated expanders to prevent implant exposure following postmastectomy radiotherapy.^27^ Valdatta et al., in their meta-analysis on the use of ADM and radiotherapy identified a prevalence of failure of reconstruction after radiotherapy of 14.05% and of skin necrosis of around 15.5%.^28^

The present study, in light of these high failure rates of heterologous breast reconstruction in irradiated patients, demonstrates that the anterior approach scarless LD muscle flap is a safe technique that does not lead to any reconstructive failure and is associated with a low capsular contracture rate.

Therefore, utilizing a dependable and easily harvestable muscle flap to cover the lower pole of the implant offers advantages in terms of capsular contracture, protection, and palpability of the implant.

**Figure 7:**
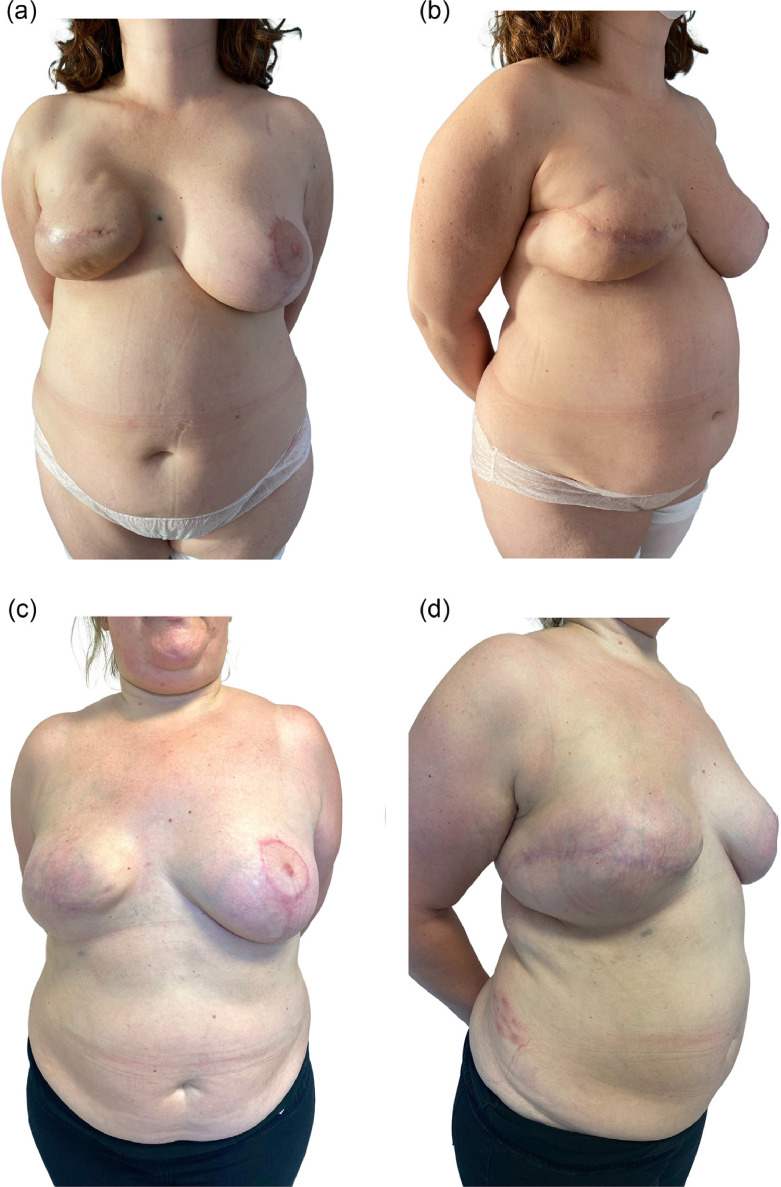
Preoperative (**a-b**) and postoperative (**c-d**) views**:** the patient underwent skin-sparing mastectomy, prepectoral reconstruction and adjuvant radiotherapy; after 18 months she developed severe capsular contracture, for which she underwent latissimus dorsi flap reconstruction.

However, the study exhibited several limitations primarily associated with its retrospective nature and a short median follow-up. Despite these limitations, it is important to note that the indications and surgical procedures outlined in this paper were standardized and consistently performed over time by the same surgeons at the same single center. Future prospective studies are essential to further validate this technique. Nonetheless, our series on anterior approach scarless LD muscle flap breast reconstruction presents intriguing data on the procedure, showcasing encouraging results in terms of safety and reliability, along with excellent reconstructive and aesthetic outcomes (Fig 7a-d).

## Conclusion

This study, in line with the literature, appears to affirm that anterior approach scarless LD muscle flap breast reconstruction is a viable option for achieving safe and cosmetically satisfactory breast reconstruction in selected patients facing complications or failures from primary surgery, or with poor soft tissue coverage. The benefits of this technique are notably pronounced in patients with a history of irradiation or those requiring postmastectomy radiation therapy, maintaining low complication and capsular contracture rates while achieving good aesthetic results. Further studies are necessary to validate these findings.

## Appendix Supplementary materials

Image, video 1
